# Advances in diagnosis of lung fibrosis: focus on present and future approaches

**DOI:** 10.3389/fmed.2026.1847987

**Published:** 2026-06-23

**Authors:** Tarig Fadelelmoula, Hamdi Al Mutori, Khalid Mohammed, Mazin Saleh, Ali Al Reesi

**Affiliations:** 1 Department of Medicine, College of Medicine and Health Sciences, National University of Science and Technology, Sohar, Oman; 2 Department of Medicine, Sohar Hospital, Sohar, North Al Batina Governorate, Oman

**Keywords:** artificial intelligence, biomarkers, diagnosis, gene expression profiling, pulmonary fibrosis, imaging, lung fibrosis, machine learning

## Abstract

**Background:**

Lung fibrosis encompasses a group of interstitial lung diseases (ILDs) characterized by progressive scarring of lung tissue, often leading to respiratory failure and high mortality. Contemporary diagnostic frameworks have evolved from diagnosis-centered to pattern-based approaches, incorporating the concept of progressive pulmonary fibrosis (PPF) as a unifying clinical entity. Accurate and timely diagnosis is critical for guiding appropriate management but remains challenging due to non-specific symptoms, overlapping radiological patterns, and limitations of existing diagnostic tools. We aimed to summarize the status, limitations, and emerging approaches in the diagnosis of lung fibrosis, with an emphasis on imaging modalities, histopathological techniques, molecular diagnostics, artificial intelligence (AI) applications, and to propose an updated diagnostic algorithm.

**Evidence and methods:**

A structured narrative review was conducted using PubMed/MEDLINE, Scopus, Web of Science, and Google Scholar to identify relevant literature published between January 2015 and February 2026. Priority was given to international clinical guidelines, consensus statements, systematic reviews, meta-analyses, and clinically relevant original studies addressing diagnostic approaches to lung fibrosis and interstitial lung diseases. The retrieved evidence was synthesized thematically, focusing on imaging modalities, histopathological techniques, molecular diagnostics, biomarkers, artificial intelligence, and multidisciplinary diagnostic frameworks.

**Results:**

High-resolution computed tomography (HRCT) remains the gold standard for non-invasive diagnosis, with pattern classification guided by the 2022 ATS/ERS/JRS/ALAT guidelines. MRI, lung ultrasound, and functional imaging offer valuable adjuncts. Surgical lung biopsy provides histopathological confirmation but carries a risk that varies depending on patient selection; transbronchial lung cryobiopsy (TBLC) has emerged as a less invasive alternative with diagnostic yields exceeding 80% in multidisciplinary settings. Emerging techniques, including gene expression profiling, telomere length assessment, circulating biomarkers, endobronchial optical coherence tomography, and AI-enhanced imaging, show promise for improving early and accurate diagnosis but remain adjuncts to multidisciplinary discussion (MDD) rather than replacements. Home-based monitoring technologies and molecular imaging have expanded capabilities for longitudinal disease monitoring. Despite these advancements, persistent challenges include diagnostic variability, limited access to advanced modalities, and the absence of standardized diagnostic algorithms.

**Conclusion:**

In summary, advances in imaging, molecular diagnostics, and artificial intelligence are improving the early and accurate diagnosis of lung fibrosis. Importantly, these tools are complementary to, not substitutes for, multidisciplinary care. Their integration into MDD-centered frameworks is essential to improve patient outcomes.

## 1 Introduction

Lung fibrosis is a pathological process characterized by excessive extracellular matrix accumulation and structural distortion of lung tissue, resulting in impaired gas exchange and progressive respiratory failure (1). It occurs across a wide range of interstitial lung diseases (ILDs), including those with known causes, such as connective tissue diseases and environmental factors, as well as idiopathic cases, notably idiopathic pulmonary fibrosis (IPF) (2). IPF is a severe, incurable form of pulmonary fibrosis, marked by irreversible fibrotic remodeling of the lung parenchyma (3). Pulmonary fibrosis often leads to high morbidity and mortality, particularly with progression despite immunosuppressive treatment, a pattern now recognized as progressive fibrosing ILD (4). The disease develops through complex interactions between epithelial injury, abnormal wound repair, fibroblast activation, and immune system dysregulation. Over the past 20 years, the primary focus has shifted from chronic inflammation to abnormal epithelial-mesenchymal interactions and fibrogenesis (5). Genetic factors, such as mutations affecting telomere maintenance and surfactant production, along with environmental and occupational exposures, influence disease risk and progression (6). IPF, the most studied subtype, is characterized by a usual interstitial pneumonia (UIP) pattern on imaging or histology and typically follows a relentless clinical course (7). Accounting for 15%−30% of ILD cases (8, 9), IPF has a median survival of 2–5 years from diagnosis (10, 11). The majority of deaths are due to respiratory failure or associated comorbidities (12, 13).

A critical advance in recent ILD nosology is the concept of progressive pulmonary fibrosis (PPF), formally introduced in the 2022 ATS/ERS/JRS/ALAT clinical practice guideline (14). PPF encompasses any ILD of known or unknown cause that exhibits progressive fibrotic behavior, manifested by worsening respiratory symptoms, declining lung function [Forced Vital Capacity (FVC) or Diffusion Capacity of Lungs for Carbon Monoxide (DLCO)], or an increased radiological extent of fibrosis, regardless of the underlying diagnosis. This concept is clinically actionable because anti-fibrotic therapy with nintedanib has demonstrated efficacy in PPF regardless of etiology (15), shifting the focus of treatment decisions toward detecting progressive fibrotic behavior as a distinct and treatable clinical phenotype, rather than relying solely on a specific ILD subtype diagnosis.

Contemporary diagnostic frameworks in ILD have increasingly adopted a pattern-based approach, distinct from the traditional diagnosis-first paradigm. This approach, reflected in emerging 2025 ERS/ATS classification trends, prioritizes identifying radiological and histopathological patterns, such as UIP, non-specific interstitial pneumonia (NSIP), organizing pneumonia, or the alveolar macrophage pattern, before assigning an underlying etiology (15). A UIP pattern may be associated with IPF, connective tissue disease, hypersensitivity pneumonitis, or drug exposure, and its diagnostic significance varies substantially depending on the clinical context. This pattern-vs.-diagnosis distinction underscores the central role of MDD in integrating pattern recognition with clinical, serological, and environmental data to arrive at a specific diagnosis.

While the etiology of some ILDs is unclear, most cases are related to factors such as genetic predisposition, environmental exposures (allergens, toxins, air pollution), autoimmune diseases (systemic sclerosis [SSc], rheumatoid arthritis, Sjögren's syndrome, and others), or certain medications (amiodarone, methotrexate, bleomycin) (16). Table 1 lists different interstitial lung diseases categorized by etiology, alongside their pathological descriptions.

**Table 1:** Interstitial lung diseases categorized by etiology and pathological description.

Disease	Pathological description
Group 1: idiopathic interstitial pneumonias (IIPs)	
Idiopathic pulmonary fibrosis (IPF)	Most common fibrotic ILD. Chronic, progressive fibrosing interstitial pneumonia of unknown cause; characterized by a UIP pattern on HRCT or histology
Non-specific interstitial pneumonia (NSIP, idiopathic)	Fibrosing ILD with temporal and spatial homogeneity on histology; aetiologically and clinically heterogeneous (CTD-associated, idiopathic, drug-induced forms)
Cryptogenic organizing pneumonia (COP)	Non-infectious pneumonia with intra-alveolar granulation tissue; may lead to fibrosis if chronic or untreated
Alveolar macrophage pneumonia (AMP)	Smoking-related ILD with diffuse alveolar macrophage accumulation; reclassified from Desquamative Interstitial Pneumonia per recent expert consensus
Pleuroparenchymal fibroelastosis (PPFE)	Rare fibrotic ILD with upper-lobe predominant fibroelastosis of the pleura and subpleural parenchyma; recognized contributor to progressive pulmonary fibrosis.
Group 2: connective tissue disease-associated ILD (CTD-ILD)	
Systemic sclerosis (SSc)-ILD	Most common CTD-ILD overall. NSIP pattern predominates; anti-Scl-70 is associated with diffuse SSc and worse ILD prognosis; anti-centromere is associated with limited SSc and milder ILD course
Rheumatoid arthritis (RA)-ILD	UIP pattern is the most common (may mimic IPF); NSIP is also seen; anti-CCP and RF are key serological markers; older male patients are at higher risk; multidisciplinary discussion is essential to distinguish it from IPF
Antisynthetase syndrome (ASS)-ILD	A distinct IIM entity. ILD is present in >70% of cases, the most common IIM-associated ILD; no longer classified merely as a PM/DM subset. Classic triad: ILD + inflammatory myopathy + mechanic's hands; fever, Raynaud's, and arthritis are common. Antibodies: Anti-Jo-1 (most common); PL-7, PL-12, EJ, OJ, KS, Ha, Zo also implicated. Histology: NSIP or OP pattern predominates
Dermatomyositis (DM)-ILD	Heterogeneous, antibody-driven subtypes. ILD risk and severity vary significantly by antibody subtype. Anti-MDA5^+^ DM-ILD: Rapidly Progressive ILD (RP-ILD) with high short-term mortality; often presents as clinically amyopathic DM (CADM). Clinical clues: skin ulceration, palmar papules, periungual erythema; myositis may be absent or mild. Requires urgent recognition and aggressive combined immunosuppression; early MDT involvement is essential. Anti-NXP2 / Anti-TIF1γ DM: ILD is less common in these subtypes. There is a strong association with malignancy, thorough cancer screening is mandatory; anti-TIF1γ is particularly linked to solid tumors
Sjögren's syndrome-ILD	LIP or NSIP pattern; anti-SSA(Ro)/SSB(La) antibodies; ILD often subclinical; lymphoma risk should be considered in all Sjögren's-ILD cases; regular surveillance recommended
Polymyositis (PM)-ILD	Per the current EULAR/ACR classification (2017), PM is now an uncommon and less well-defined IIM subtype. Many historical PM-ILD cases likely represent misclassified Antisynthetase Syndrome or IMNM. Diagnosis requires exclusion of other IIM subtypes; ILD association is less frequent than previously reported
Group 3: exposure-related ILD	
Hypersensitivity pneumonitis (HP)	Immune-mediated ILD due to repeated inhalation of environmental antigens; fibrotic HP may be difficult to distinguish from IPF
Pneumoconiosis	Occupational lung diseases from inhalation of mineral dusts; silicosis characterized by upper- and mid-lobe small, rounded nodules and progressive massive fibrosis (PMF); asbestosis is characterized by pleural plaques and basal fibrosis; coal worker's pneumoconiosis is characterized by upper-lobe nodules
Radiation-induced pulmonary fibrosis	Fibrotic reaction following radiation therapy, localized to the radiation field on imaging
Group 4: drug-induced ILD	
Drug-induced pulmonary fibrosis	Caused by medications including amiodarone, methotrexate, bleomycin, and cyclophosphamide. Immune checkpoint inhibitors (ICIs), including PD-1 inhibitors pembrolizumab and nivolumab and PD-L1 inhibitor atezolizumab, are now among the most common causes of drug-induced ILD in oncology practice
Group 5: granulomatous ILD	
Sarcoidosis	Granulomatous disease with non-caseating granulomas on biopsy; HRCT shows upper-lobe fibrosis and lymphadenopathy
Pulmonary langerhans cell histiocytosis (PLCH)	Smoking-related ILD; HRCT shows upper-lobe cysts and nodules; CD1a+ Langerhans cells on biopsy
Group 6: eosinophilic ILD	
Chronic eosinophilic pneumonia	Inflammatory lung disease with peripheral eosinophilia; BAL eosinophilia >25%; responds to corticosteroids; potential fibrosis if persistent
Group 7: unclassifiable and secondary fibrosis	
Unclassifiable ILD (uILD)	Fibrotic ILD is not categorized after a comprehensive MDD evaluation; it represents up to 10–15% of ILD cases at referral centers
Post-ARDS/ALI fibrosis	Fibrotic sequelae following acute lung injury or ARDS are an increasingly recognized cause of progressive pulmonary fibrosis

ALI, acute lung injury; ARDS, acute respiratory distress syndrome; ASS, antisynthetase syndrome; BAL, bronchoalveolar lavage; CADM, clinically amyopathic dermatomyositis; COP, cryptogenic organizing pneumonia; CTD-ILD, connective tissue disease-associated ILD; DM, dermatomyositis; HP, hypersensitivity pneumonitis; HRCT, high-resolution CT; ICI, immune checkpoint inhibitor; IIM, idiopathic inflammatory myopathy; ILD, interstitial lung disease; IMNM, immune-mediated necrotising myopathy; IPF, idiopathic pulmonary fibrosis; LIP, lymphoid interstitial pneumonia; MDT, multidisciplinary team; NSIP, non-specific interstitial pneumonia; OP, organizing pneumonia; PLCH, pulmonary langerhans cell histiocytosis; PM, polymyositis; PMF, progressive massive fibrosis; PPFE, pleuroparenchymal fibroelastosis; RA, rheumatoid arthritis; RF, rheumatoid factor; RP-ILD, rapidly progressive ILD; SSc, systemic sclerosis; uILD, unclassifiable ILD; UIP, usual interstitial pneumonia.

The non-specific clinical presentation, typically characterized by exertional dyspnea and dry cough, combined with a broad differential diagnosis, makes accurate and timely diagnosis of lung fibrosis a significant challenge (17). Early recognition is crucial; delays can lead to missed opportunities to initiate disease-modifying treatments or to enroll patients in clinical trials (18). Diagnosis is especially difficult for idiopathic pulmonary fibrosis, which mimics other interstitial lung diseases and lacks sensitive early detection methods (19). Despite advances in imaging and multidisciplinary approaches, limitations persist. Multidisciplinary discussions among pulmonologists, radiologists, and pathologists remain the gold standard for accurate diagnosis (20, 21).

## 2 Evidence and methods

This narrative review was conducted using a structured and transparent literature search strategy to provide a balanced synthesis of current and emerging diagnostic approaches for lung fibrosis, with a particular focus on interstitial lung diseases (ILDs) and idiopathic pulmonary fibrosis (IPF).

### 2.1 Search approach

Electronic databases, including PubMed/MEDLINE, Scopus, Web of Science, and Google Scholar, were searched for relevant English-language articles published between January 2015 and February 2026. Additional key references were identified by manually screening the reference lists of selected articles, clinical guidelines, and consensus statements. Search terms included “lung fibrosis,” “pulmonary fibrosis,” “interstitial lung disease,” “idiopathic pulmonary fibrosis,” “progressive pulmonary fibrosis,” “high-resolution computed tomography,” “usual interstitial pneumonia,” “transbronchial lung cryobiopsy,” “surgical lung biopsy,” “biomarkers,” “KL-6,” “MMP-7,” “gene expression profiling,” “telomere length,” “artificial intelligence,” “machine learning,” and “multidisciplinary discussion.”

### 2.2 Study selection

Priority was given to international clinical guidelines, expert consensus statements, systematic reviews, meta-analyses, randomized controlled trials, large observational studies, and clinically relevant original research. Foundational studies were included when necessary to explain established diagnostic concepts, historical context, or widely accepted diagnostic frameworks.

### 2.3 Eligibility criteria

Inclusion criteria

The inclusion criteria were as follows:

Studies addressing diagnostic approaches for lung fibrosis, ILDs, IPF, or progressive pulmonary fibrosis.
Articles evaluating imaging, histopathological, biomarker-based, molecular, genetic, artificial intelligence, or machine learning diagnostic methods.
Studies discussing multidisciplinary diagnosis, diagnostic algorithms, or clinical decision-making in fibrotic ILD.
Peer-reviewed English-language publications with clear clinical relevance.

### 2.4 Exclusion criteria

The exclusion criteria were as follows:

Case reports, small case series, editorials, and non-peer-reviewed sources.
Studies focused primarily on treatment without diagnostic relevance.
Articles lacking sufficient methodological clarity, clinical applicability, or relevance to fibrotic ILD diagnosis.
Non-English publications where reliable interpretation was not possible.

### 2.5 Data synthesis

Evidence was synthesized thematically according to major diagnostic domains: imaging, histopathology, molecular diagnostics, circulating biomarkers, artificial intelligence and machine learning, home-based monitoring, and future diagnostic strategies. Emphasis was placed on diagnostic utility, limitations, accessibility, validation status, and integration into multidisciplinary discussion-based diagnostic pathways. Areas of consensus, uncertainty, and emerging innovations were highlighted to support the proposed diagnostic framework.

## 3 Status of present diagnostic approaches

Diagnostic approaches for lung fibrosis integrate imaging, histopathology, and multidisciplinary discussion (MDD). High-resolution computed tomography (HRCT) is the primary diagnostic tool, while tissue sampling techniques and novel imaging modalities provide supplementary support in selected cases. Despite these advances, current methods are constrained by diagnostic uncertainty, interobserver variability, procedural risks, and limited sensitivity for early disease detection.

### 3.1 Present imaging techniques

High-resolution Computed Tomography (HRCT) is now the key diagnostic tool, providing vital structural details without invasive procedures. It is especially effective at detecting the usual interstitial pneumonia (UIP) pattern, a hallmark of IPF (22). However, radiological findings can sometimes be unclear, and variability among clinicians, even at expert centers, remains high (23).

The 2022 ATS/ERS/JRS/ALAT guideline and the Fleischner Society White Paper define four HRCT pattern categories that standardize clinical decision-making in ILD (14, 24). A Typical UIP pattern, honeycombing with or without peripheral traction bronchiectasis, subpleural and basal predominant, permits a confident IPF diagnosis in the appropriate clinical context without surgical lung biopsy. A probable UIP pattern is subpleural and basal predominant reticulation with traction bronchiectasis, without honeycombing, supporting an IPF diagnosis through MDD when other causes have been excluded; biopsy may be considered when diagnostic uncertainty remains. An indeterminate for UIP pattern includes subtle reticulation or mild ground-glass opacity that does not meet the criteria for other diagnoses and requires further evaluation. An alternative diagnosis pattern, upper- or mid-lung predominant fibrosis, peribronchovascular distribution, or extensive ground-glass opacity, should prompt evaluation for HP, CTD-ILD, or other conditions. Applying these four categories consistently is essential for standardizing multidisciplinary team communication and for appropriately triaging patients for lung biopsy.

Magnetic resonance imaging (MRI) is becoming an increasingly important tool for assessing lung fibrosis, offering a non-invasive, radiation-free alternative to HRCT, particularly for patients requiring serial imaging or those at risk of radiation exposure (25). MRI has demonstrated potential in detecting fibrotic lung changes through specialized techniques (26). T2-weighted MRI sequences highlight tissues with high water content and can visualize lung parenchymal abnormalities linked to IPF and other fibrosing ILDs (27). Building on this, ultrashort echo time (UTE) MRI greatly reduces the time between the radiofrequency pulse and signal detection. UTE MRI has shown the ability to differentiate fibrotic lesions from malignancies in complex cases, such as progressive massive fibrosis (28).

Functional imaging techniques such as magnetic resonance elastography and diffusion-weighted imaging (DWI) further enhance diagnostic capabilities by assessing tissue stiffness and fibrotic progression (29). While MRI remains less spatially resolved than HRCT, its advantages in tissue characterization, especially with dynamic or contrast-enhanced sequences, make it a promising tool for monitoring fibrosis and detecting early disease (25). Elastic registration of inspiratory-to-expiratory MRI has proven useful in detecting pulmonary fibrosis in systemic sclerosis by measuring lung deformation, showing high diagnostic accuracy compared with CT (30).

Lung ultrasound (LUS) is increasingly recognized as a valuable, radiation-free, bedside imaging modality for detecting and monitoring pulmonary fibrosis (31). It depends on identifying sonographic artifacts such as B-lines, pleural irregularities, and line disruptions—features associated with fibrotic interstitial lung diseases (32). Recent studies show that LUS can reliably correlate with HRCT findings (33–35). In idiopathic pulmonary fibrosis (IPF), pleural line thickening and a B-line count ≥3.0 have achieved high diagnostic accuracy, with B-lines showing a strong correlation with CT findings (36, 37). Quantitative ultrasound spectroscopy further improves diagnostic precision; frequency-based analysis of B-lines achieved a sensitivity and specificity of up to 92% in differentiating pulmonary fibrosis from other causes of B-lines (38). Additionally, a simplified LUS protocol effectively identified honeycombing and reticulation patterns typical of fibrosis, with high interobserver agreement across specialties (26). LUS offers a sensitive, non-invasive, and rapid alternative for evaluating lung fibrosis and may serve as a frontline tool for screening and monitoring, especially where HRCT is not feasible or contraindicated (39).

### 3.2 Present histopathological techniques

Surgical lung biopsy (SLB) remains a crucial tool for diagnosing fibrotic ILDs, particularly when non-invasive clinical and radiological evaluations are inconclusive (40). SLB provides histopathologic confirmation that can critically inform diagnosis and treatment (41). SLB is most beneficial for patients with uncertain diagnoses or atypical imaging features. When clinical history and HRCT strongly suggest IPF, SLB may be unnecessary, with up to 96% diagnostic accuracy achieved in expert centers without tissue biopsy (42). SLB enhances diagnostic confidence significantly in fibrotic ILDs and remains the standard when alternative diagnoses are under consideration (43, 44).

Transbronchial lung cryobiopsy (TBLC) has emerged as the preferred bronchoscopic approach for tissue sampling in fibrosing ILD. When integrated into an MDD framework, TBLC achieves diagnostic yields of 80%−90%, with studies demonstrating diagnostic concordance with SLB in appropriately selected patients (45, 46). Unlike conventional transbronchial forceps biopsy, TBLC uses cryoprobes to obtain larger, better-preserved tissue samples, enabling reliable histopathological classification of UIP, NSIP, and HP. The 2022 ATS/ERS/JRS/ALAT guideline endorses TBLC as an acceptable alternative to SLB in centers with appropriate expertise and safety protocols (14). Its safety profile, particularly in experienced centers, compares favorably with that of SLB, making TBLC the bronchoscopic biopsy method of first choice in fibrosing ILD where tissue sampling is required.

Transbronchial lung biopsy (TBLB) using flexible forceps has limited utility in fibrotic ILD due to the small sample size obtained and the predominantly peripheral, subpleural distribution of fibrosis, which is not reliably accessible via this technique. TBLB carries a high non-diagnostic rate in UIP-pattern disease and is not recommended as the primary bronchoscopic sampling method for fibrotic ILD (14). Its role is appropriately restricted to conditions with bronchocentric pathology, such as sarcoidosis and organizing pneumonia, and to settings where TBLC is unavailable. In a study excluding patients with classical IPF radiologic patterns, TBLB achieved an 85.7% diagnostic yield in a selected non-IPF cohort (47); however, this finding should not be extrapolated to its general utility in fibrotic ILD.

Thoracoscopic biopsy using a Vicryl loop safely and efficiently obtains lung tissue in pediatric patients, minimizing operative time and complications. It is viable for children who qualify for surgical intervention and lack contraindications, but not for those with severe respiratory compromise or poor surgical candidacy (48). Multidisciplinary discussions among pulmonologists, radiologists, and pathologists are the gold standard for diagnosing IPF and other fibrosing ILDs, endorsed by major guidelines to enhance diagnostic precision and guide treatment (20, 21).

### 3.3 Limitations of present diagnostic methods

Conventional imaging modalities (CT, MRI, and ultrasound) perform poorly at detecting early-stage fibrosis. Functional methods such as MR and US elastography show promise but remain limited by technical artifacts and access (49). SLB, while definitive, is invasive, unsuitable for many patients due to comorbidities and procedural risks, and is also affected by sampling error and inter-observer variability (50). The perioperative risk of SLB is context-dependent and should not be stated as a uniform figure. In patients with multiple comorbidities and advanced disease, aggregate risk scores have estimated 30-day mortality as high as 11.5% (51); however, in carefully selected patients at experienced referral centers, contemporary series report 30-day mortality of approximately 1.7%, confirming that SLB is a safe procedure when patient selection is rigorous (42, 50). Key modifiers of procedural risk include age, baseline FVC and DLCO, the presence of pulmonary hypertension, and overall frailty. SLB can also lead to transient declines in lung function; in one study, FVC declined by an average of 156 mL within 6 months post-biopsy, though outcomes were worse in patients with already impaired lung function (52). TBLC is increasingly preferred as a less invasive alternative to SLB in eligible patients, offering a more favorable risk-benefit profile. Multidisciplinary discussions enhance diagnostic precision and guide appropriate treatment, but they are resource-intensive (20, 21). A summary of present lung fibrosis diagnostic methods is presented in Table 2.

**Table 2:** Comparison of strengths and weaknesses of present lung fibrosis diagnostic methods.

Diagnostic method	Strengths	Weaknesses
High-resolution CT	High spatial resolution; gold standard for detecting hallmark fibrosis patterns; guides four-tier UIP classification (typical, probable, indeterminate, alternative)	Radiation exposure; operator-dependent interpretation; less sensitive to early disease
Magnetic resonance imaging	Radiation-free; assesses structural and functional changes; useful in pediatric or repeat assessments	Lower spatial resolution than CT; expensive; less available in clinical settings
Ultrasound	Safe, portable, and inexpensive; detects peripheral fibrosis and subpleural abnormalities	Limited to lung periphery; operator-dependent; cannot visualize deep lung tissue
Surgical lung biopsy (SLB)	Gold standard for histopathological diagnosis; allows detailed classification of fibrosis pattern	Highly invasive; 30-day mortality ranges from ~1.7% (carefully selected patients) to ~11.5% (high-risk cohorts); risk modified by age, FVC, DLCO, and pulmonary hypertension
Transbronchial lung cryobiopsy (TBLC)	Diagnostic yield 80–90% with MDD; larger, better-preserved tissue samples than TBLB; endorsed by 2022 ATS/ERS/JRS/ALAT guideline; preferred bronchoscopic approach in fibrotic ILD	Risk of bleeding and pneumothorax; requires an experienced center; not widely available
Transbronchial lung biopsy (TBLB)	Minimally invasive; accessible; useful in bronchocentric conditions (sarcoidosis, organizing pneumonia)	Small sample size; high non-diagnostic rate in fibrotic ILD; low yield in peripheral fibrosis; not recommended as primary tool for fibrotic ILD
Thoracoscopic biopsy with vicryl loop	Yields large, high-quality samples; effective for subpleural fibrosis; viable in pediatric settings	Invasive; surgical risks; not suitable for frail patients
Multidisciplinary discussion (MDD)	Integrates radiological, clinical, and pathological data; improves diagnostic consensus and accuracy; gold standard endorsed by international guidelines	Resource-intensive; may not be feasible in all healthcare settings; depends on expert availability

## 4 Challenges in the diagnosis of lung fibrosis

Diagnosing lung fibrosis is difficult because many ILDs share similar clinical, radiological, and histopathological features. Delayed patient presentation, limited access to specialized multidisciplinary teams, and inconsistent diagnostic methods further contribute to diagnostic uncertainty. Invasive procedures are often unsuitable for patients who are older, frail, or have significant comorbidities. The following sections summarize the main challenges to achieving an accurate and timely diagnosis.

### 4.1 Non-specific symptoms

Lung fibrosis often presents with non-specific symptoms such as dry cough and dyspnea, leading to frequent misdiagnosis and delays in specialist referral (53).

### 4.2 Radiological and histological overlap

High-resolution CT (HRCT) patterns, such as usual interstitial pneumonia (UIP), can also be seen in other conditions, including hypersensitivity pneumonitis and connective tissue diseases, complicating diagnosis (54). The four-tier UIP classification (typical, probable, indeterminate, alternative) partially addresses this overlap but does not eliminate diagnostic uncertainty, particularly in the indeterminate category, where clinical and histopathological integration through MDD is required (14, 24).

### 4.3 Risk of invasive procedures

Surgical lung biopsies should be considered in patients with either clinical or CT findings that are indeterminate for IPF, but many patients are too frail or elderly to undergo the procedure safely (24). TBLC offers a less invasive alternative with high diagnostic yield in appropriate settings, but access to experienced centers remains uneven (45, 46).

### 4.4 Multidisciplinary complexity

Diagnosis relies heavily on multidisciplinary discussion among pulmonologists, radiologists, and pathologists, which may not be available in all settings (55).

### 4.5 Lack of standardized algorithms

Diagnostic criteria vary across institutions and practitioners, and a lack of universal standards contributes to diagnostic variability and uncertainty (56). The absence of widely adopted, validated diagnostic algorithms that integrate emerging biomarker and molecular data further compounds this challenge.

## 5 Emerging molecular diagnostic techniques

Emerging molecular diagnostic approaches offer the potential to refine and supplement pattern-based ILD diagnosis. However, most of these methods remain largely investigational and have not yet been validated for widespread routine clinical use. Their role, at present, is to support and enrich the MDD process rather than to replace it.

### 5.1 Gene expression profiling (GEP)

GEP identifies molecular signatures associated with fibrosis pathogenesis, activity, and subtypes, thereby enhancing diagnostic precision in selected settings, and can differentiate IPF from other ILDs and may help classify histologically ambiguous cases (57). Spatial gene expression analysis has revealed distinct transcriptional patterns across fibrotic lung regions in IPF, chronic HP, and NSIP, supporting reclassification of unclassifiable ILD cases in research settings (58). Whole-genome expression studies in peripheral blood have identified over 1,000 genes whose expression correlates with IPF severity, offering potential prognostic biomarkers (59). Comparative profiling has identified distinct transcriptional profiles in IPF and NSIP, supporting their classification as distinct conditions (60). One target, Twist1, has been shown to promote fibroblast survival and proliferation in fibrotic lungs (61). Key limitations restrict GEP's clinical use: assay platforms are not standardized, costs are high, and prospective multicenter validation is lacking (57). The Envisia genomic classifier is among the few GEP applications designed to detect UIP in transbronchial biopsies (62), but its use is limited to select referral centers and lacks universal guideline endorsement. Clinicians should use GEP results to support MDD decision-making, and not as standalone diagnostic tools.

### 5.2 Telomere length measurement (TLM)

TLM is a clinically relevant biomarker for diagnosing and characterizing pulmonary fibrosis, especially IPF and familial interstitial pneumonia (FIP). Telomeres are protective caps at the ends of chromosomes that shorten with age and with each cell division. Critically short telomeres drive fibrotic lung disease. Up to 25% of patients with sporadic IPF and 40% of familial cases have abnormally short telomeres, regardless of age or smoking status (63). This dysfunction leads to alveolar epithelial cell aging, faulty repair, and increased fibrotic changes (64). Measuring telomere length in blood leukocytes through qPCR or flow-FISH is a non-invasive way to identify patients at genetic risk. Short telomeres are associated with earlier disease onset, faster progression, and more complications after immunosuppression or lung transplantation (65). Mutations in telomerase or other telomere-related genes also help guide familial disease screening and refine diagnosis in atypical or unclassifiable ILD (66). Important caveats apply: technical variability exists across qPCR and flow-FISH platforms, population-level reference ranges are not universally standardized, and TLM is not currently endorsed for routine use in non-familial ILD by major guidelines (63, 66). Its greatest clinical utility at present lies in the investigation of familial ILD and atypical presentations where a genetic predisposition is suspected.

### 5.3 Circulating biomarkers

Circulating biomarkers are a non-invasive approach for diagnosing and monitoring lung fibrosis, especially IPF and related ILDs (67). These biomarkers reflect pathophysiological processes such as epithelial injury, extracellular matrix remodeling, and immune dysregulation (68). However, circulating biomarkers primarily reflect disease activity and fibrosis burden and generally lack the specificity to classify ILD subtypes; they are most appropriately used as adjuncts to MDD-based diagnosis and for disease monitoring.

The most studied markers are KL-6, surfactant proteins A and D (SP-A, SP-D), and matrix metalloproteinase-7 (MMP-7). KL-6, a glycoprotein secreted by regenerating alveolar type II cells, shows sensitivity and specificity above 80% for diagnosing ILD in appropriate clinical settings (67). However, KL-6 is not specific to ILD and may also be elevated in pulmonary hypertension, lung malignancy, and other pulmonary conditions (69). Standardized KL-6 assays are mainly available in Japan, parts of Europe, and the Asia-Pacific region. SP-A and SP-D, produced by alveolar type II cells, reflect lung injury and inflammation and are primarily used as adjuncts for monitoring rather than for diagnosis. MMP-7, a protease involved in extracellular matrix remodeling, is associated with disease progression and outcomes in IPF. Its sensitivity and specificity for distinguishing IPF from non-IPF ILD are reported at 80–85%, but it is not widely used outside research settings (69).

Circulating fibrocytes, derived from bone marrow, are involved in fibrogenesis. Elevated levels in fibrotic lung diseases correlate with impaired lung function and mortality, indicating potential as prognostic markers (70).

Circulating microRNAs are emerging as biomarkers for differentiating fibrotic from non-fibrotic lung states (71). However, reproducibility across laboratories is limited, and no standardized assay protocols for microRNAs or circulating fibrocytes have been endorsed by regulatory bodies (70, 71).

Carbohydrate antigen (CA19-9) has been investigated as an adjunct biomarker in ILD; elevated levels have been reported in IPF and CTD-ILD in observational studies, and some evidence suggests a correlation with HRCT fibrosis extent (72). However, CA19-9 has substantially lower ILD specificity than KL-6 or MMP-7, given its well-established elevation in pancreatic, biliary, and other gastrointestinal malignancies, and it is therefore considered of investigational interest rather than a first-line diagnostic biomarker. All circulating biomarker results should be interpreted within the broader MDD context.

### 5.4 Endobronchial optical coherence tomography (EB-OCT)

EB-OCT is a non-destructive imaging modality that enables high-resolution, real-time imaging of lung microanatomy via bronchoscopy. Its minimally invasive nature allows it to be performed immediately prior to SLB, demonstrating comparable diagnostic performance without the risks of surgical procedures (73). Despite these promising preliminary results and exciting possible applications, additional robust data are needed to better understand the utility and limitations of OCT before widespread clinical implementation (74). EB-OCT should therefore be regarded as a promising adjunct for selected cases rather than a standard diagnostic step.

## 6 Machine learning-based diagnostic techniques

Advances in machine learning, artificial intelligence, molecular imaging, and digital health technologies are transforming the diagnosis of fibrotic interstitial lung diseases, especially idiopathic pulmonary fibrosis. However, most of these approaches remain experimental and require further prospective multicenter validation before broad clinical adoption.

### 6.1 Image-based machine learning (ML)

Image-based ML has demonstrated high accuracy in identifying IPF and UIP (75, 76). A 2025 meta-analysis reported that ML models achieved a diagnostic C-index (concordance index) of 0.93 for IPF and 0.91 for UIP, significantly outperforming radiologists in sensitivity while maintaining comparable specificity (77). A recent study demonstrated ML-assisted polarization-sensitive OCT for *in vivo* assessment of lung fibrosis with 77% accuracy and 89% specificity, potentially reducing reliance on invasive biopsies (78). Advanced ML techniques, such as semi-supervised learning, have been applied to hierarchical phase-contrast tomography for vessel segmentation, aiming to detect early fibrotic changes before conventional imaging can visualize them (79). A graph-based ML model applied to HRCT outperformed three experienced radiologists in identifying fibrotic ILD, achieving an AUC of 0.973 and an external validation accuracy of 98.6% (80). These results warrant cautious interpretation pending independent prospective multicenter validation.

### 6.2 ML from metabolic and hematological data

In addition to imaging, ML models trained on complete metabolic profile (CMP) and complete blood count (CBC) accurately classified IPF and predicted disease severity with discriminatory values exceeding 0.74, offering complementary diagnostic pathways (81).

### 6.3 Hybrid deep learning and clinical data models

Hybrid deep learning models using HRCT and clinical data achieved 95% diagnostic accuracy for IPF, outperforming multidisciplinary diagnosis in development cohorts (82). These results suggest strong potential for clinical decision support but require prospective multicenter validation before clinical deployment.

### 6.4 Fibresolve

Fibresolve is a machine learning system designed for clinical diagnosis of IPF by analyzing 3D chest CT scans to identify patterns associated with IPF, including those beyond the classic UIP pattern. It showed high sensitivity and specificity even in cases without definitive UIP patterns, indicating its promise for early, non-invasive diagnosis of IPF (83, 84).

### 6.5 Molecular imaging

Molecular imaging techniques, such as positron emission tomography with computed tomography (PET/CT), are promising approaches for detecting and characterizing pulmonary fibrosis (85). Unlike traditional imaging, which primarily captures structural alterations, PET/CT assesses active fibrogenesis, making it helpful for evaluating disease progression and response to anti-fibrotic treatments (86, 87).

### 6.6 Home-based monitoring devices

Home-based monitoring devices are now common in managing lung fibrosis, especially IPF and fibrosing ILDs (85). Their primary validated function is disease monitoring and follow-up rather than initial diagnosis (88). These tools, spirometers, pulse oximeters, and mobile health apps, allow patients to track lung function and symptoms remotely. Home spirometry is feasible, produces reliable data that match hospital-based results, and patients report high satisfaction and adherence (89). Emerging data suggest that continuous home physiological monitoring may eventually contribute to earlier identification of subclinical deterioration in at-risk populations; however, this diagnostic application remains investigational and is not yet validated for primary diagnostic use (90). Accordingly, home monitoring tools are positioned in this manuscript within the post-diagnosis monitoring pathway rather than within the initial diagnostic algorithm.

### 6.7 Limitations of AI and machine learning approaches

Despite promising diagnostic performance, several important limitations must be acknowledged before AI and ML tools can be considered for routine clinical implementation. The majority of published models were trained on data from single or a few referral centers, with datasets that over-represent IPF relative to rarer ILD subtypes, limiting generalisability to community settings (75). Although many models demonstrate high accuracy on internal validation, performance declines on independent external datasets, a manifestation of overfitting, and high AUC values reported in individual studies have not been consistently reproduced in independent multicenter validation (77). Many deep learning models function as “black boxes,” providing limited mechanistic insight into diagnostic outputs; explainability approaches such as gradient-weighted class activation mapping are active areas of development but not yet standardized (80). Finally, all AI tools must be framed as decision-support adjuncts within MDD frameworks rather than independent diagnostic systems.

## 7 Future approaches in the diagnosis of lung fibrosis

Looking ahead, the most impactful advances are likely to come not from individual technologies in isolation but from their integration into standardized, accessible, and MDD-supported diagnostic workflows. The following subsections describe the anticipated translational trajectory of the key approaches.

### 7.1 Biomarkers

The next frontier in ILD biomarker development is the deployment of multiplexed biomarker panels combining KL-6, MMP-7, SP-D, and novel candidates (e.g., lysophosphatidic acid, YKL-40) into integrated risk-stratification tools. Miniaturization technologies are expected to enable point-of-care platforms, facilitating earlier triage prior to HRCT in primary care and low-resource settings (91).

### 7.2 Genomics

Ongoing development of validated genomic classifiers for transbronchial biopsy specimens, particularly for distinguishing UIP from non-UIP patterns without surgical biopsy, represents a significant near-term opportunity. Integration of polygenic risk scores incorporating common genetic variants (e.g., MUC5B promoter variant, TOLLIP) with clinical risk factors may enable population-level risk stratification and earlier surveillance in genetically predisposed individuals (62).

### 7.3 Machine learning

Federated learning frameworks, allowing ML models to be trained across multiple institutions without sharing patient data, offer a practical pathway to address dataset bias and improve generalizability. Prospective implementation trials and real-world evidence studies are needed to generate the outcome data required for regulatory approval and guideline endorsement of AI-assisted diagnostic tools (92).

### 7.4 Molecular imaging

Novel PET tracers targeting collagen synthesis, integrin activation, and fibroblast-specific markers are in active development and may allow real-time, non-invasive quantification of active fibrogenesis *in vivo*, enabling disease activity monitoring and treatment response assessment with greater specificity than structural imaging alone (93).

### 7.5 Telemedicine and AI-integrated remote monitoring

AI-enhanced integration of home spirometry, pulse oximetry, and patient-reported outcome data into predictive algorithms may transform reactive monitoring into proactive early-warning systems for acute exacerbations and disease progression. Ensuring equitable access to these platforms across healthcare systems, including low- and middle-income settings, remains a critical implementation challenge (94).

## 8 Proposed diagnostic algorithm

The diagnostic strategy for lung fibrosis starts with a detailed clinical assessment. This includes a history of environmental exposures, comorbidities, and symptoms. Pulmonary function testing helps detect restrictive patterns. HRCT is key to identifying characteristic imaging patterns. The four-tier UIP classification guides the diagnostic pathway: typical, probable, indeterminate for UIP, or alternative diagnosis (14, 24). MDD is required at each decision point. It integrates clinical, radiological, and histopathological data, including for ILDs with identifiable causes. Serologic testing and exposure history are needed to rule out secondary causes (16). Molecular and genetic testing (63–66) and minimally invasive biopsy techniques, especially TBLC, the preferred bronchoscopic tissue-sampling approach (45, 46), are recommended when diagnostic uncertainty exists. Table 3 lists hallmark diagnostic criteria for common ILDs.

**Table 3:** Interstitial lung disease and its updated diagnostic criteria in different tests.

Interstitial lung disease	Diagnostic criteria
Idiopathic pulmonary fibrosis (IPF)	HRCT showing: (1) Typical UIP pattern, honeycombing ± traction bronchiectasis, subpleural and basal predominant; or (2) Probable UIP pattern, subpleural and basal predominant reticulation with traction bronchiectasis, without honeycombing. Absence of identifiable cause; both patterns require integration through MDD
Non-specific interstitial pneumonia (NSIP)	HRCT with ground-glass opacities, fine reticulation, subpleural sparing, and minimal honeycombing; histopathology with temporally and spatially uniform interstitial fibrosis; commonly associated with CTD
Hypersensitivity pneumonitis (HP)	HRCT with mosaic attenuation, air-trapping; history of antigen exposure; BAL lymphocytosis
Connective tissue disease-associated ILD (CTD-ILD)	Autoantibodies: ANA, anti-CCP antibodies (preferred over RF for RA-ILD due to higher specificity), anti-Scl-70 (SSc), myositis-specific antibodies (anti-Jo-1, anti-MDA5 for IIM-ILD); HRCT with NSIP or UIP pattern; systemic signs of autoimmune disease
Sarcoidosis	Non-caseating granulomas on biopsy; HRCT with upper-lobe fibrosis and lymphadenopathy
Pneumoconiosis	History of occupational dust exposure; silicosis: upper and mid-lobe small, rounded nodules and progressive massive fibrosis (PMF); asbestosis: basal fibrosis and pleural plaques; coal worker's pneumoconiosis: upper-lobe nodules
Drug-induced pulmonary fibrosis	Temporal relationship with drug use (including chemotherapy agents, amiodarone, methotrexate, or immune checkpoint inhibitors); HRCT showing diffuse infiltrates; exclusion of other causes
Radiation-induced pulmonary fibrosis	History of radiation; fibrosis confined to the radiation field on imaging
Cryptogenic organizing pneumonia (COP)	HRCT with patchy peripheral consolidations; histopathology showing intra-alveolar buds of granulation tissue
Chronic eosinophilic pneumonia	Peripheral infiltrates on imaging; peripheral eosinophilia; BAL eosinophilia >25%; response to steroids
Pulmonary Langerhans cell histiocytosis	HRCT with cysts and nodules in the upper lobes; CD1a+ Langerhans cells on biopsy
Alveolar macrophage pneumonia (AMP, formerly DIP)	HRCT with ground-glass opacities; biopsy showing alveolar macrophages filling airspaces
Unclassifiable ILD (uILD)	Fibrotic ILD pattern on HRCT and/or histology; comprehensive MDD evaluation fails to yield a specific diagnosis

**Figure 1:**
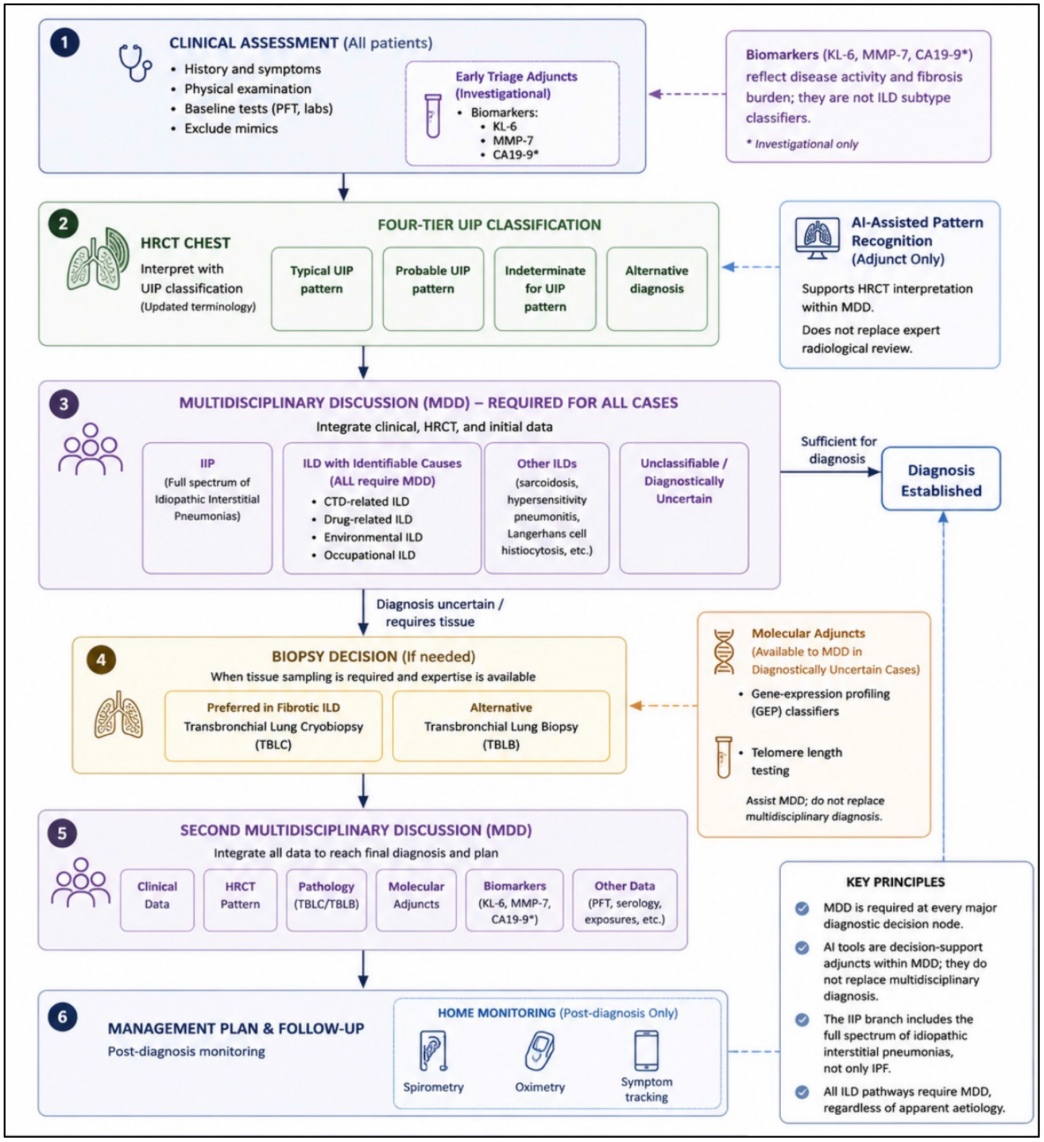
Proposed diagnostic algorithm for lung fibrosis using current and emerging methods.

Novel diagnostic tools are used at specific decision points in the algorithm (Figure 1). Circulating biomarkers such as KL-6 and MMP-7 help with early triage during the initial assessment and may speed up referral for HRCT (69). AI-assisted HRCT pattern recognition tools assist radiologists (82). These tools are especially helpful in cases with probable or indeterminate UIP patterns, where objective fibrosis quantification can improve MDD. GEP-based classifiers and telomere length testing are available to MDD teams when HRCT and clinical data are not enough for diagnosis (57). Biomarkers and home monitoring technologies are used after diagnosis for disease surveillance (89–91). Their role is to monitor, not to perform primary diagnosis. These tools help reduce diagnostic uncertainty at specific points in the MDD process. They complement multidisciplinary diagnosis, not replace it.

## 9 Conclusion

Recent advances have unified and improved lung fibrosis diagnostics by anchoring pattern-based clinical frameworks within multidisciplinary approaches. HRCT now provides the gold-standard, four-tier UIP pattern classification, supported by functional imaging and safer tissue-sampling methods, such as transbronchial lung cryobiopsy. Molecular diagnostics and machine learning enhance but do not replace clinical evaluation, as their roles remain largely investigational. Home monitoring technologies extend surveillance but serve as complements to, rather than substitutes for, initial clinical evaluation.

The central principle is clear: emerging technologies, AI, molecular diagnostics, biomarkers, and telemedicine are adjuncts, not replacements for the multidisciplinary team. Multidisciplinary discussion remains the diagnostic gold standard in ILD. Effective integration of these tools within multidisciplinary care, with an emphasis on validation, accessibility, and equity, enables earlier intervention and better outcomes.

## 10 Review limitations

As a narrative synthesis, this review does not provide quantitative comparisons or a formal risk-of-bias assessment. The included literature reflects publications available up to the search date and therefore may not capture all emerging evidence in this rapidly evolving field. Furthermore, the applicability of novel diagnostic technologies, including AI models, molecular diagnostic tests, and advanced imaging modalities, may vary considerably across healthcare settings due to differences in resource availability, regulatory frameworks, institutional expertise, and patient population demographics. These limitations should be considered when interpreting the generalizability of the proposed diagnostic framework.
